# British Adolescents Are More Likely Than Children to Support Bystanders Who Challenge Exclusion of Immigrant Peers

**DOI:** 10.3389/fpsyg.2022.837276

**Published:** 2022-08-08

**Authors:** Seçil Gönültaş, Eirini Ketzitzidou Argyri, Ayşe Şule Yüksel, Sally B. Palmer, Luke McGuire, Melanie Killen, Adam Rutland

**Affiliations:** ^1^Department of Psychology, University of Exeter, Exeter, United Kingdom; ^2^Department of Psychology, Bilkent University, Ankara, Turkey; ^3^Graduate School of Education, University of Exeter, Exeter, United Kingdom; ^4^Department of Human Development and Quantitative Methodology, University of Maryland, College Park, MD, United States

**Keywords:** moral reasoning, evaluation of a challenger, group functioning, intergroup and intragroup social exclusion, immigrants

## Abstract

The present study examined British children’s and adolescents’ individual and perceived group evaluations of a challenger when a member of one’s own group excludes a British national or an immigrant newcomer to the school (Turkish or Australian) from participating in a group activity. Participants included British children (*n* = 110, *M_age in years_* = 9.69, *SD* = 1.07, 44 girls, aged 8–11) and adolescents (*n* = 193, *M_age in years_* = 14.16, *SD* = 0.92, 104 girls, aged 13–16), who were inducted into their group and heard hypothetical scenarios in which a member of their own group expressed a desire to exclude the newcomer from joining their activity. Subsequently, participants heard that another member of the ingroup challenged the exclusionary act by stating that they should be inclusive. Children’s and adolescents’ individual evaluations of the bystander who challenged the social exclusion of an immigrant peer were more positive than their perceived group evaluations, recognizing that groups are often exclusionary. Only adolescents but not children differed in their individual and perceived group evaluations in the social exclusion of British peers. When the newcomer was an immigrant peer, adolescents were more likely to evaluate the challenger positively in both their individual and perceived group evaluations compared to children. Further, children, compared to adolescents, were more likely to reason about social and group norms to justify their evaluations only when the excluded peer was an immigrant but not when the excluded peer was British. Adolescents were more likely to reason about fairness, rights, and equality. The findings indicate that exclusionary group norms surrounding immigrants begin in childhood. Interventions that focus on changing group norms to be more inclusive could be effective in reducing prejudicial attitudes toward immigrants in childhood.

## Introduction

Immigrant children and adolescents represent a growing part of the United Kingdom population (Vargas-Silva and Rienzo, 2019; Office for National Statistics, 2020). As a result, intergroup interactions between immigrant and non-immigrant children and adolescents are becoming increasingly likely in schools in the United Kingdom. However, despite this increase in intergroup interactions, immigrant children and adolescents are at higher risk of experiencing social exclusion because of their national identity (Stevens et al., 2020). As bystanders are central actors who can stop intergroup social exclusion when they challenge the excluder, it is critically important to understand how non-immigrant children and adolescents consider peer group members who stand up against the social exclusion of immigrant peers (Evans et al., 2014; Palmer and Abbott, 2018). Termed a “challenger,” peers who stand up to members of their group that victimize and harass others are a central factor in reducing prejudice and changing group norms. Thus, investigating how children and adolescents evaluate and reason about bystanders who challenge exclusionary behaviors and treatment is both urgent and timely.

Social exclusion includes both intergroup (e.g., when an outgroup member is excluded) and intragroup (i.e., when an ingroup member is excluded) contexts (Killen et al., 2013a). In the current study, we examined British children’s and adolescents’ individual evaluations, and their perception of their group’s evaluation of a challenger who stands up against the social exclusion of immigrant (intergroup) and British (intragroup) peers.

### The Social Reasoning Developmental Perspective

The present study examined children’s and adolescents’ individual and perceived group evaluation of challenger based on the premises of the social reasoning developmental (SRD) model (Killen and Rutland, 2011; Rutland and Killen, 2015). Children and adolescents often experience incidents that require them to make decisions about whom to include in, or exclude from, their peer activities within their daily lives. The SRD model provides a theoretical and empirical framework to examine children’s and adolescents’ behaviors, attitudes, and reasoning in such situations by integrating the social domain theory (SDT, Turiel, 1983; Smetana et al., 2014) and social identity development and group dynamics theories (Nesdale, 2004; Abrams and Rutland, 2008). By drawing on these theories, the SRD model enables to understand how children and adolescents reason about fairness, equality, and concerns for others to challenge social exclusion and to understand how they attribute group functioning, social and group norms while justifying their exclusionary attitudes and behaviors (Killen and Rutland, 2011).

Empirical studies based on the SRD model have also indicated that in some contexts, children and adolescents evaluate social exclusion as unacceptable based on the unfair treatment of others (Palmer et al., 2015; Mulvey et al., 2016). As well, they also support instances of social exclusion for reasons related to group functioning and group dynamics (Hitti et al., 2011; Mulvey, 2016). Further, the SRD model proposes that, with age, children become more capable of balancing moral and group concerns when evaluating social exclusion by recognizing the multifaceted nature of it (Killen and Rutland, 2011; Rutland and Killen, 2015). As a complex process, social exclusion can occur at many levels including intragroup and intergroup, and it is highly likely to occur covertly (Rutland and Killen, 2015). Thus, examining how children and adolescents reason about their peers’ approach (e.g., challenging or supporting) toward intragroup and intergroup social exclusion provides a stage for researchers to understand interpretations and motivations that underlie exclusionary behavior and treatments in different contexts. Unlike *intragroup* social exclusion (Killen and Malti, 2015), *intergroup* social exclusion is mostly rooted in prejudice, discrimination, and negative attitudes toward the targeted outgroups. Examining and comparing children’s and adolescents’ evaluation of challenger peers in different social exclusion contexts will provide insights into the developmental awareness of the role that intergroup processes play when evaluating and reasoning about how ingroup and outgroup members respond to social exclusion and victimization. It is vital to understand these processes to identify the ways to promote inclusive schools, especially in intergroup contexts (Palmer et al., 2021).

### Individual and Group Evaluations of a Peer Who Challenges Social Exclusion

Although earlier research has focused on excluded peers of social exclusion and excluders, there has been a recent shift to focus on bystanders. Bystanders, who are peers witnessing, social exclusion, and other different types of victimization can serve as central actors to offset both the occurrence and effects of social exclusion and other types of peer aggression (Salmivalli et al., 2011). Research in the area of *intragroup* exclusion reveals that when bystanders challenge social exclusion and bullying, these incidences tend to cease within a short time (Hawkins et al., 2001; Salmivalli et al., 2011). Yet, research has not fully delved into the role of bystanders for intergroup social exclusion.

Children and adolescents are concerned with fairness and often act prosocially to challenge someone being unfairly socially excluded in both intragroup and intergroup contexts (Killen and Rutland, 2011). Yet, especially in intergroup contexts when deciding whether to challenge social exclusion, individuals must also consider group norms and how their group will react to an ingroup peer who challenges social exclusion.

For example, the national identity of the excluded peer might shape how children and adolescents evaluate an ingroup member who challenges the exclusion including whether the excluded peer is from the same group as the child doing the excluding (e.g., non-immigrant peer) or from an outgroup (e.g., immigrant peer; Palmer et al., 2022). Further, understanding children’s and adolescents’ cognition and reasoning about the role of the bystander and the potential costs involved of challenging exclusionary behavior sheds light on the interpretations and motivations that underlie responses to victimization.

A growing literature on bystander responses to social exclusion has revealed that children and adolescents have differentiated judgments about the likelihood that a member of their own group would challenge an act of aggression committed by a member of their own group. Further, studies have shown that children and adolescents differentiate their own judgments from the groups’ judgments in intergroup settings involving stigmatization or status (Mulvey et al., 2014, 2018; Mulvey and Killen, 2016). For example, Mulvey and Killen (2016) showed that children (9 to 10-year-olds) and adolescents (13 to 14-year-olds) were individually more supportive of challenges to peer aggression than they expected their group to be in a gender-based intergroup context. Similarly, both children and adolescents were more likely to report that they would be more supportive of challenger peers than their group would in the context of challenging gender stereotypes (Mulvey and Killen, 2016). Youth recognize that there is a cost to challenging group norms even when they view the challenging act as legitimate and sometimes imperative as in the case of bullying and harassment.

The differentiation between children’s and adolescents’ own judgments and their perception about their group’s judgments has also been found in different intergroup contexts. For example, Mulvey et al. (2018) showed differentiation in individual and group judgments in a social inclusion context in which they manipulated language spoken by outgroup members (e.g., Spanish, Chinese, or Arabic speaking). They found that children (aged 8–11 years) were more likely to rate their own inclusivity judgments of a language-outgroup member as higher compared to their group’s inclusivity judgments documenting differentiation between their own perspective and their group’s perspective. Thus, youth recognize that group norms apply to the ingroup and the outgroup.

### Age-Related Differences in Individual Versus Group Evaluations of the Challenger

Drawing on the SRD model (Rutland et al., 2010; Killen and Rutland, 2011) the ability to differentiate between individuals’ own perspective and their group’s perspective can be important for social interactions in which there is a need to consider multiple perspectives. By late childhood individuals typically evaluate exclusion in intergroup contexts negatively, though they perceive their group may be less negative about such exclusion. Age differences regarding this distinction have been documented in different intergroup contexts. For example, McGuire et al. (2019) found that adolescents’ (13- to 5-year-olds) ability to differentiate between their own evaluation and group perspective in an inter-school context was more stable as compared to children (8- to 11-year-olds). Similarly, in a gender-based intergroup context, participants (aged between 9.5 and 13.5 years) differentiated their own individual favorability from the group’s favorability for an ingroup challenger as they get older (Mulvey et al., 2014). Together these studies show how the interaction between context and age impacts children’s and adolescents’ individual and group evaluation of the challenger. It is particularly important to examine individual evaluations together with perceived group evaluations across different age groups considering the importance of peer influence in children’s and adolescents’ decision-making in social exclusion.

To our knowledge, no studies have examined age-related changes regarding evaluations and reasoning about whether a peer would challenge as a bystander, their individual evaluations of challenging, and their perception of their group’s evaluation of challenging social exclusion of immigrant peers. Youth’s judgments and reasoning about the group processes surrounding bystander challenging are important, as understanding these social cognitions may ultimately help reduce prejudice-based social exclusion.

### Present Study

The current study examined British children’s and adolescents’ individual evaluations, their perception of their group’s evaluations, and reasoning about the challenger of the social exclusion of immigrant (either Turkish or Australian) and British peers by drawing from the SRD approach to social exclusion (Rutland et al., 2010; Killen and Rutland, 2011; Rutland and Killen, 2015). Further, we examined whether the difference between an individual’s evaluation of a challenger peer and their perception of their group’s evaluation of an ingroup challenger was present in intergroup and non-intergroup contexts. It is important to note that the current study is part of a larger project that examines bystander judgments and responses to the intergroup social exclusion of immigrants.

In the current study, participants were presented with hypothetical scenarios of either non-immigrant (British) or immigrant peers (Turkish or Australian). Both groups were reported to be newcomers as they had recently moved to the school featured in the scenarios; the distinction was that the British youth moved from another area in Britain and the immigrants moved to the United Kingdom from their home country. We purposefully chose immigrants as the intergroup context because immigrants are one of the groups stigmatized and treated differently in the United Kingdom based on different characteristics nationality, religion, and language (Ford et al., 2015; Creighton and Jamal, 2020). Considering the widespread and long-lasting effects of social exclusion on immigrant youth (psychological well-being, physical health, educational attainment), it is critically important to identify how children and adolescents evaluate their peers’ challenging behaviors to create inclusive norms in school contexts (Oxman-Martinez et al., 2012; Rodríguez Hidalgo et al., 2014). For explanatory purposes, we also manipulated the nationality of the immigrant being excluded, so they were either a Turkish immigrant peer or an Australian immigrant peer in the scenarios. Although different immigrant groups in the United Kingdom share common experiences (e.g., moving from another country), each of these immigrant groups might have unique characteristics and might be perceived differently by British individuals. Thus, we also examined whether British children’s and adolescents’ evaluations differ when their ingroup members challenge the social exclusion of immigrants from different backgrounds.

As a summary, in the current study, we examined both participants’ individual evaluations and their perceptions of group evaluations of the challenger to gain a more comprehensive understanding of bystanders’ judgments in intergroup contexts in relation to group dynamics. Further, we also examined our participants’ reasoning about their evaluations of the challenger of social exclusion to have insight into what drives their motivation in their evaluations (fairness, prejudice, discrimination, group norms, societal conventions, etc.) based on the SDT (Turiel, 1983).

The following hypotheses were tested:

### Hypotheses

Participants’ individual and group evaluations of the challenger peer were expected to differ based on the exclusion condition (whether excluded peer was an immigrant peer vs. a British peer) and age (children and adolescents):

1. We expected that participants would be more likely to evaluate the challenger’s action as acceptable in intragroup (when the excluded peer is British) compared to intergroup (when the excluded peer is an immigrant) social exclusion.
2. Similarly, we hypothesized that participants would be more likely to think that their group would evaluate the challenger act as more okay in intragroup (when the excluded peer is British) compared to intergroup (when the excluded peer is an immigrant) social exclusion.
3. We expected that both adolescents and children would report that their group would evaluate the challenger less positively than they would in intergroup social exclusion of immigrants as group identities and norms should become more salient in this condition.
4. We expected that adolescents but not children would differentiate between their individual and group evaluation in intragroup social exclusion when the excluded peer was a British peer as adolescents are cognitively able to attend to what a group might expect better in both intergroup and intragroup context.

Participants’ reasoning for their judgments were expected to differ based on their evaluations:

5. We expected that reasoning justifications would differ based on participants’ individual and group evaluation of the challenger (okay or not okay).

Age and condition base differences in participants’ reasoning judgments were also examined for exploratory purposes.

## Materials and Methods

### Participants

Our initial sample consisted of 386 participants including 133 children (*M_age in years_* = 9.67, *SD* = 1.08, 57 girls, aged 8–11) and 253 adolescents (*M_age in years_* = 14.23, *SD* = 0.94, 135 girls, aged 13–16). We excluded participants who did not identify themselves as British (*no* = 42; *I do not know* = 11). Participants who failed to answer attention check questions about where their own group of friends (*n* = 22) and the excluded peer (*n* = 17) were born were also dropped from analyses. Overall, the final sample included 110 children (*M_age in years_* = 9.69, *SD* = 1.07, 44 girls, aged 8–11) and 193 adolescents (*M_age in years_* = 14.16, *SD* = 0.92, 104 girls, aged 13–16). The ethnic breakdown of our final sample was as follows: White-British (71%), White-European (10.6%), White-Irish (3%), White-Polish (0.3%), Bangladeshi, Indian or Sri Lankan (2%), Black-Caribbean (0.3%), mixed-race (3.4%), or “other” (9.6%). The G*Power analysis (alpha of 0.05, power of 0.95, and an effect size of 0.25) demonstrated that 279 participants were required (Faul et al., 2007).

### Design

Our original design was 2 (Age group: children and adolescents) x 3 (Exclusion condition: Turkish, Australian, British) between-participant design. However, as we did not find differences between the two immigrant conditions in our dependent variables (Turkish and Australian), we merged those into one category called as immigrant condition (explained in detail in the data analysis section below). The dependent variables were participants’ individual and group evaluations of a challenger peer’s bystander reaction to the social exclusion, and participants’ reasoning responses to their individual and group evaluations.

### Procedure

After obtaining Ethics Committee Board approval, we introduced the study to the school principals. All participants were recruited by sending invitation letters and consent forms to parents through their headteachers. Both parental consent and participants’ own assent were sought. All students with parental consent who assented to participate were included in the study. Participants completed questionnaires online *via* survey software Qualtrics. Participants worked on their own computers, within class-sized groups, with support from trained researchers where needed. Debriefs were provided verbally (to participants) and in writing *via* letters sent home to primary caregivers. Small gifts (e.g., stickers or pens) were given to participants as a token of thanks for their participation.

### Measures

#### Initial Group Affiliation Story

Participants were presented with the following initial group manipulation scenario: “*We would like you to imagine that you are in the story and tell us what you think of what is happening. In the story, let us say that you are part of a group of friends who all live in England, which is in Britain. All your friends in this group were born here in Britain. Everyone in this group describes themselves as British”* (based on the previous literature, e.g., Killen et al., 2013a; Mulvey and Killen, 2016; Mulvey et al., 2016). This hypothetical friendship group description was accompanied by gender-matched silhouettes of a group of friends (see Supplementary Documents for the gender-matched silhouettes). A question (“Where were your friends in this story born?”) was asked as a comprehension check question. Participants who failed to answer were dropped from the analyses (*n* = 22). After they were introduced to the group, they completed a brief group affiliation task to increase shared identity with the group. For this task, they selected a name and a symbol for their group. Participants were also asked to rate the following question “How much do you like being part of this group of British friends? (1 = *no way*, 6 = *yes, definitely*)” to see whether the affiliation task worked. Descriptive statistics showed that overall participants reported that they liked being part of this group (Turkish exclusion condition: *M* = 5.14, *SD* = 0.80; Australian exclusion condition: *M* = 5.07, *SD* = 0.79; British exclusion condition: *M* = 5.06, *SD* = 1.00) and no significant differences were found between conditions (all *ps* > 0.05). Then, participants were asked to imagine their group of friends had chosen to go to an after-school cooking and baking club, “that involves cooking and baking food that is popular in Britain.”

#### Social Exclusion Story

After the group affiliation part, participants read about a newcomer to the school (described as Turkish or Australian or British): “Imagine one week there’s a new student who has come along to your group’s cooking club and wants to join in. *Deniz/Charlie/Jamie* was born in Turkey/Australia/Britain.” Those in the Turkish/Australian conditions then read: “*Deniz/Charlie* recently moved from Turkey/Australia with his/her family to live in Britain.” Those in the British condition read: “*Jamie* recently moved here with his/her family from somewhere else in Britain.” A comprehension check ensured participants understood where the newcomer was from. Those who answered incorrectly were dropped from the analyses (*n* = 17). Participants then read that someone in their British group of friends did not want the newcomer to join (from hereon, the “excluder”): “Sam, who is in your group of friends, says to [newcomer], ‘We do not want you to join our group because you are from somewhere else - you are different’.”

### Evaluation to Challenger Reactions to Social Exclusion

After the social exclusion scenario participants read that someone in their British group of friends disagreed with excluding the newcomer (from hereon, the “challenger”): “Alex is one of the friends in your British group. They disagree with [excluder]. Alex thinks that your group should invite [newcomer] to cook with them.” After participants were asked to evaluate two outcome variables (1) individual evaluation of challenger (2) group evaluation of challenger:

#### Individual Evaluation of Challenger

To measure participants’ evaluation of challenger response, participants read the following sentences “Imagine that Alex (challenger) tells Sam (excluder) that they think the group should invite *Deniz/Charlie/Jamie* (excluded) to cook with them. How OK or not OK was it for Alex (challenger) to say that to Sam (excluder)?” and were asked to evaluate on a six-point Likert type scale (1-*definitely not OK* to 6-*yes, definitely OK*).

#### Perceived Group Evaluation of Challenger

To measure perceived group evaluations, we asked, “How OK or not OK does your group think Alex (challenger) is for telling Sam (excluder) that *Deniz/Charlie/Jamie* (excluded) should be invited to cook with the group?” Participants responded on a 1 (*definitely not OK*) to 6 (*yes, definitely OK*) scale.

#### Reasoning

After each evaluation question, participants were asked, *why do you think that?* and typed their open-ended response into a text box. Participants’ responses were coded based on a framework derived from Social Domain Theory (SDT, Turiel, 1983; Killen et al., 2013b; Smetana et al., 2014). SDT explains how individuals identify and evaluate different domains of social knowledge when judging socially relevant actions including moral (i.e., involves reasoning around issues of fairness, equality, welfare, prejudice, and discrimination), societal (i.e., relate to reasoning around social norms, group identity, group norms, and group functioning), and personal domains (i.e., involves concerns around autonomy; Turiel, 1983).

Our coding system consisted of seven categories in three different domains. In the moral domain, there was: (1) Fairness and Individual Rights, (2) Prejudice and Equality, (3) Welfare. In the social conventional domain, there was (4) Social and Group Norms, (5) Group Dynamics and Functions, (6) Repercussions and Representation Management. Finally, in the personal domain, there was (7) Autonomy. Responses that did not make sense or fell outside of these categories were coded as (8) Undifferentiated. Codes that were used less than 10% were combined conceptually with other categories of higher usages (see Table 1 for the frequencies). Each response was coded under one of those categories (no double codes were used). Interrater reliability was assessed based on 25% of the interviews, with all Cohen’s κ = 0.93.

**Table 1:** Frequencies (percentages) and examples for the reasoning.

	Individual evaluation of challenger	Perceived group evaluation of challenger
Fairness and Individual Rights	13.7% (Because Jamie deserves to be in the group just as well as everyone else)	9.4% (Because other people in the group also thinks that it’s just unfair to exclude someone)
Prejudice and Equality	7.2% (People should not be discriminated for where they come from)	3.6% (There’s nothing wrong with you or your group cooking with someone of different race)
Welfare	27.1% (Because he is standing up for Sam which makes him feel more welcome)	22.7% (Because it was her first day she needs to feel welcomed by the group)
Social and Group Norms	9.2% (it depends on what the rest of the group thinks as well)	19.4% (Because they all class themselves as British and do not want someone different joining them)
Group Dynamics and Functions	14.7% (Group could have had something planned for only that amount of people)	28.1% (Because some people in the group do not like Jamie)
Repercussions and Representation Management	1% (Because he is putting his friendship in risk as they could go against him too)	1.1% (Because they probably agree with Alex but are too scared to be “different”)
Autonomy	21.2% (Because she is expressing her opinion)	7.9% (It is her choice to say that that and no one can judge her for it)
Undifferentiated	5.8% (Because he gets to cook with them)	7.9% (There’s nothing wrong with Charlie)

For each outcome, different categories were merged and used based on their frequencies. For the “why” question related to the individual evaluation of challenger, four different categories emerged: 1-*Fairness, Rights, Prejudice, and Equality*, 2-*Welfare (of others)*, 3-*Social and Group Norms*, *Group Dynamics/Functions Repercussions and Reputation Management* and 4-*Autonomy*. For the “why” question related to group evaluation of challenger, four different categories emerged: 1-*Fairness, Rights, Prejudice, and Equality*, 2-*Welfare (of others)*, 3-*Social and Group Norms,* and 4-*Group Dynamics/Functions Repercussions and Reputation Management*.

### Data Analytic Plan

Data analysis was conducted in multiple steps. A dummy code of Turkish (−1), Australian (−1), and British (+2) was created to understand whether participants’ evaluations of the challenger and their reasoning varied based on the immigration status of the excluded peer (Exclusion condition: immigrant versus British). First, a mixed ANOVA was conducted to investigate age group and exclusion condition-based differences in participants’ individual and group evaluations. To evaluate participants’ reasoning, Multinomial Logistic Regressions were conducted to examine the relationship between our reasoning categories and our independent variables while simultaneously controlling for how each of these may be influenced by the other variables.

## Results

### Individual and Perceived Group Evaluations of Challenger

A 2 (Evaluation of challenger: individual, perceived group) x 2 (Exclusion condition: immigrant, British) x 2 (Age group: children, adolescents) repeated measures ANOVA was conducted, with individual and group evaluations of the challenger as within-participant and condition and age as between-participant factors. Results showed that there were no significant differences in participants’ individual evaluation [*F* (1, 294) = 0.46, *p* = 0.500, *η_p_*^2^ = 0.002] and perceived group evaluation [*F* (1, 294) = 0.13, *p* = 0.715, *η_p_*^2^ = 0.000] across exclusion conditions. Our H1 and H2 (main effect of exclusion condition) were not supported.

**Figure 1:**
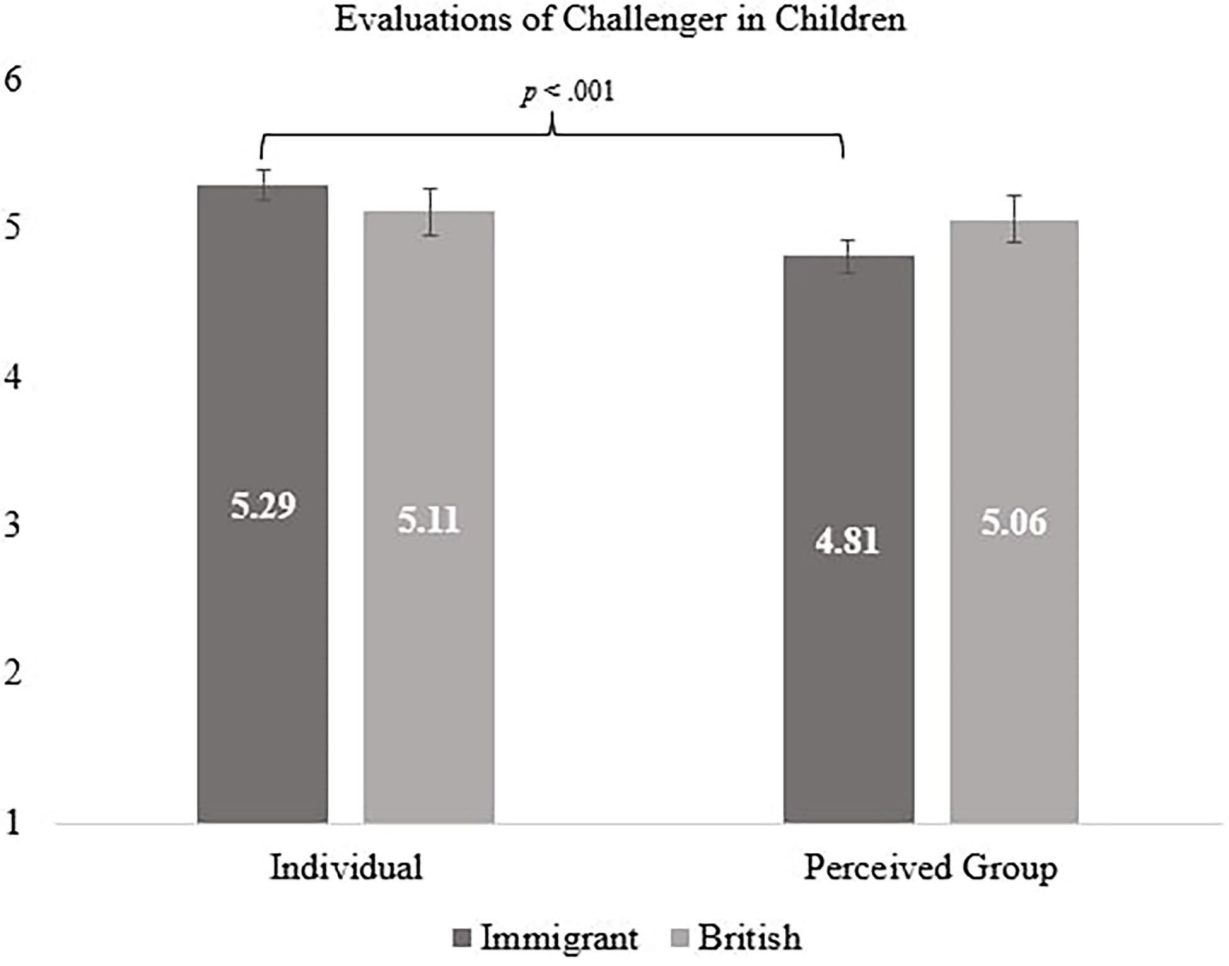
Children’s individual and perceived group evaluations of challenger by exclusion condition.

**Figure 2:**
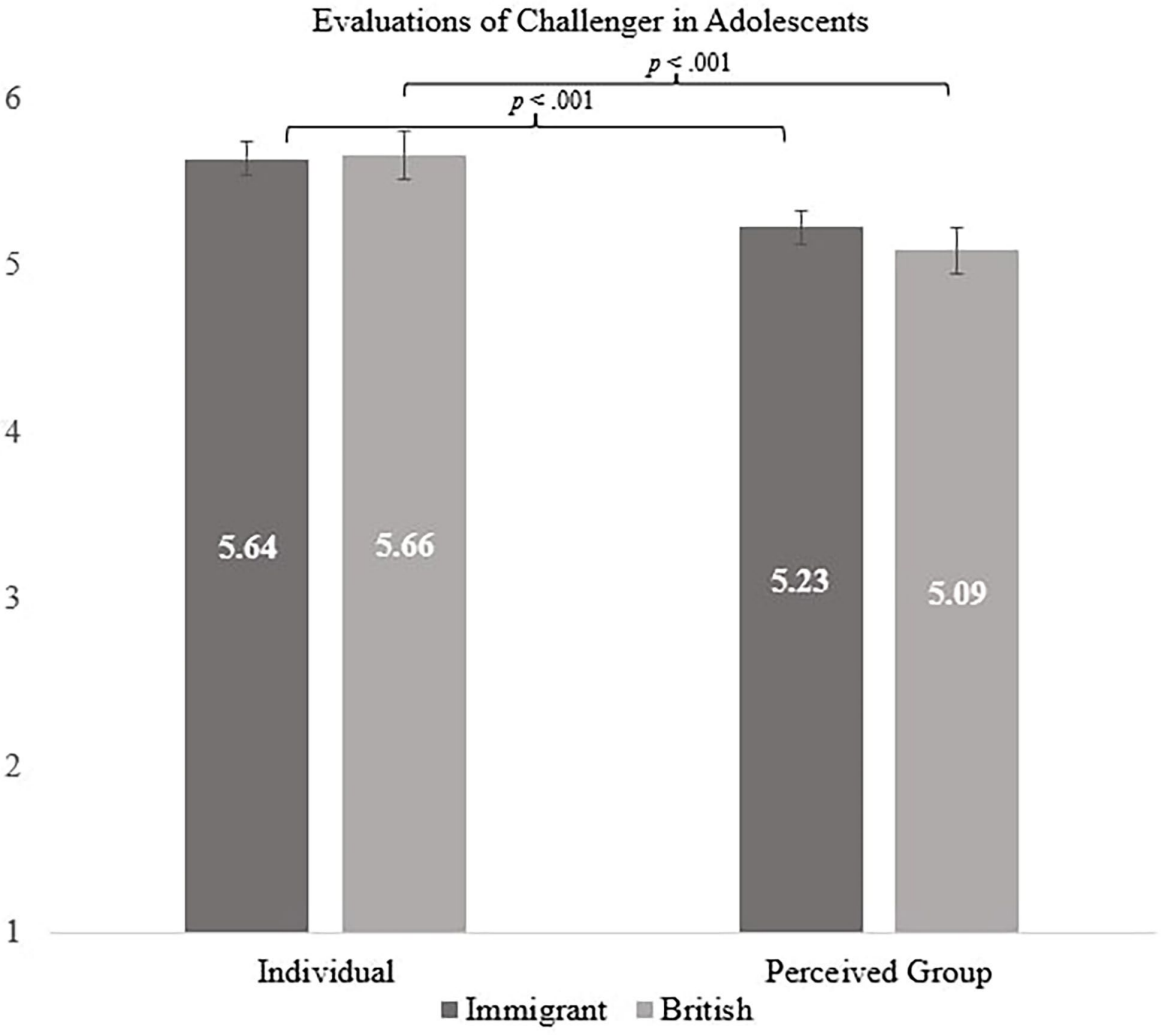
Adolescents’ individual and perceived group evaluations of challenger by exclusion condition.

However, a significant interaction between exclusion condition, age group and evaluation was found, *F* (1, 294) = 4.06, *p* = 0.045, *η_p_*^2^ = 0.014. In the immigrant exclusion condition, both children’s and adolescents’ individual evaluations of challenger were more positive than their perceived group evaluations [children: *F* (1, 294) = 13.87, *p* < 0.001, *η_p_*^2^ = 0.045; adolescents: *F* (1, 294) = 18.14, *p* < 0.001, *η_p_*^2^ = 0.058; H3 was supported]. However, in the British exclusion condition children’s individual and perceived group evaluations did not differ, while adolescents’ individual evaluations were positive compared to group evaluations [children: *F* (1, 294) = 0.09, *p* = 0.762, *η_p_*^2^ = 0.000; adolescents: *F* (1, 294) = 15.72, *p* < 0.001, *η_p_*^2^ = 0.051; Please see Figures 1, 2; supports H4].

Further, we also explored pairwise comparisons (with Bonferroni corrections) that examine children and adolescents across two outcomes (individual evaluation and perceived group evaluation of challenger). In the immigrant exclusion condition, adolescents were more likely to evaluate the challenger positively in both their individual [*F* (1, 294) = 6.78, *p* = 0.010, *η_p_*^2^ = 0.023] and perceived group evaluations [*F* (1, 294) = 6.63, *p* = 0.011, *η_p_*^2^ = 0.022] compared to children. However, in the British exclusion condition, adolescents’ individual evaluations were more positive than children’s [*F* (1, 294) = 7.66, *p* = 0.006, *η_p_*^2^ = 0.025], but children’s and adolescents’ perceived group evaluations of the challenger did not significantly differ, *F* (1, 294) = 0.01, *p* = 0.907, *η_p_*^2^ = 0.000.

### Reasoning

#### Individual Evaluations of Challenger Reasoning

Multinomial logistic regression was used to explore participants’ reasoning while justifying their individual evaluations of the challenger as a dependent variable with the following four categories: 1-Fairness, Rights, Prejudice, and Equality, 2-Welfare (of others), 3-Social and Group Norms, Group Dynamics/Functions Repercussions and Reputation Management and 4-Autonomy. Exclusion condition (immigrant and British), age groups (children and adolescents), and individual evaluation of challenger (categorical: okay versus not okay) were entered as factors. The model represents a significant improvement in fit over the null model with the addition of predictors, *χ*^2^ (9, *N* = 275) = 30.46, Nagelkerke *R*^2^ = 0.11, *p* < 0.001. Both Pearson’s chi-square test [*χ*^2^ (12) = 8.20, *p* = 0.770] and Deviance chi-square [*χ*^2^ (12) = 9.55, *p* = 0.656] indicate good fit.

**Figure 3:**
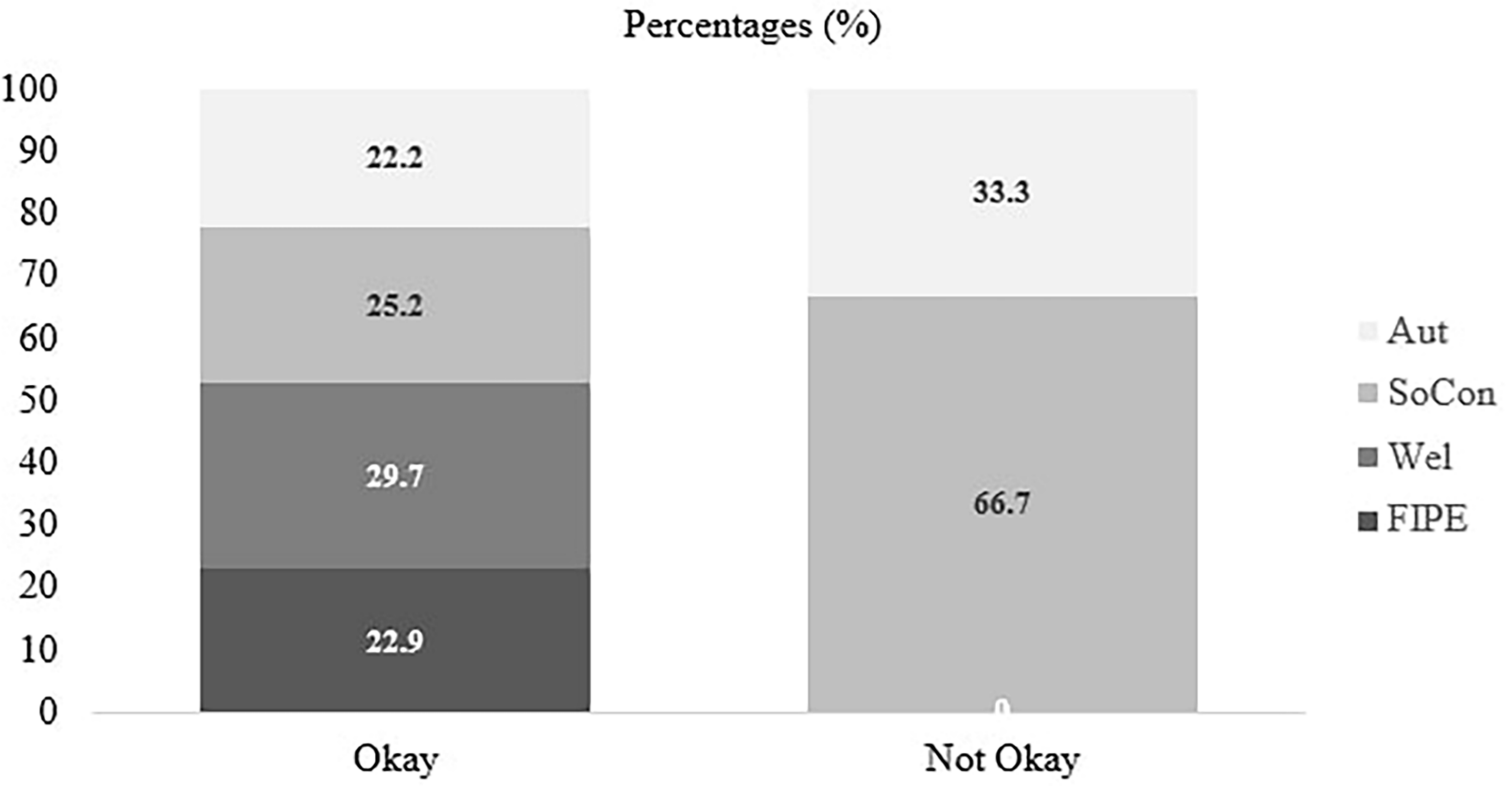
Percentages of participants reasoning of individual evaluation of challenger by individual evaluation (okay, not okay). FIPE, Fairness and Individual Rights and Prejudice and Equality; Wel, Welfare; SoCon, Social and Group Norms, Group Dynamics and Functions and Repercussions and Representation Management; and Aut, Autonomy. Numbers represent the percentages of participants within “okay” and “not okay” categories.

Result showed that individual evaluation of challenger (okay versus not okay) was found as a significant factor [*χ*^2^ (3, *N* = 275) = 13.87, *p* = 0.003]. Participants who evaluated the challenger’s actions as not okay were more likely to attribute “social and group norms” than “fairness, rights and equality” (*p* < 0.001) and “welfare” (*p* < 0.001) compared to participants who evaluated challenger as okay (H5 was supported, see Figure 3 for the raw percentages for each category across “okay” and “not okay”). Those who evaluated the challenger’s action as not okay were more likely to justify their evaluations with reference to the social norms and group functioning (e.g., “it is not okay because it will affect their friendship”) while those who evaluated the challenger’s action as okay were more likely to refer to the moral domain using fairness and welfare reasoning (e.g., “because she deserves to be treated the same as the others”; “Alex has given Jamie an opportunity to make friends and not be alone”).

A significant main effect of age was also found, *χ*^2^ (3, *N* = 275) = 11.16, *p* = 0.011. Welfare (e.g., “To make him happy”) justifications were more likely to be used than “fairness, rights and equality” (e.g., “Because Sam was being really unfair and impolite to Jamie”) justifications by children compared to adolescents (*p* = 0.009). Further, children (compared to adolescents) were more likely to attribute “social and group norms” (e.g., He is not in our group; they have to work as a group; I think that because it is always good to make new friends; Jaime was not born in the same part that the group of friends were born in) justifications relatively to “fairness, rights and equality” (e.g., “because Sam needs to know and learn that you cannot treat people differently based on where they are from; Because Charlie has just the much right as anyone else to join the group”) justifications (*p* = 0.006).

Although no significant main effect of exclusion condition was observed (immigrant vs. British), an interaction between condition and age was found, *χ*^2^ (9, *N* = 275) = 20.69, *p* = 0.014. Accordingly, children were more likely to attribute “social and group norms” than “fairness, rights and equality” only in immigrant exclusion condition, *p* = 0.025 (not in the British exclusion condition, *p* = 0.729). For example, children in the immigrant exclusion condition provided justifications like “it would be hard because Sam (excluder) is your friend and because the group might not need her, and they do not know what she is like yet.” Contrary to children, adolescents’ justifications about “social and group norms” and “fairness, rights, and equality” did not differ from each other in both conditions.

**Figure 4:**
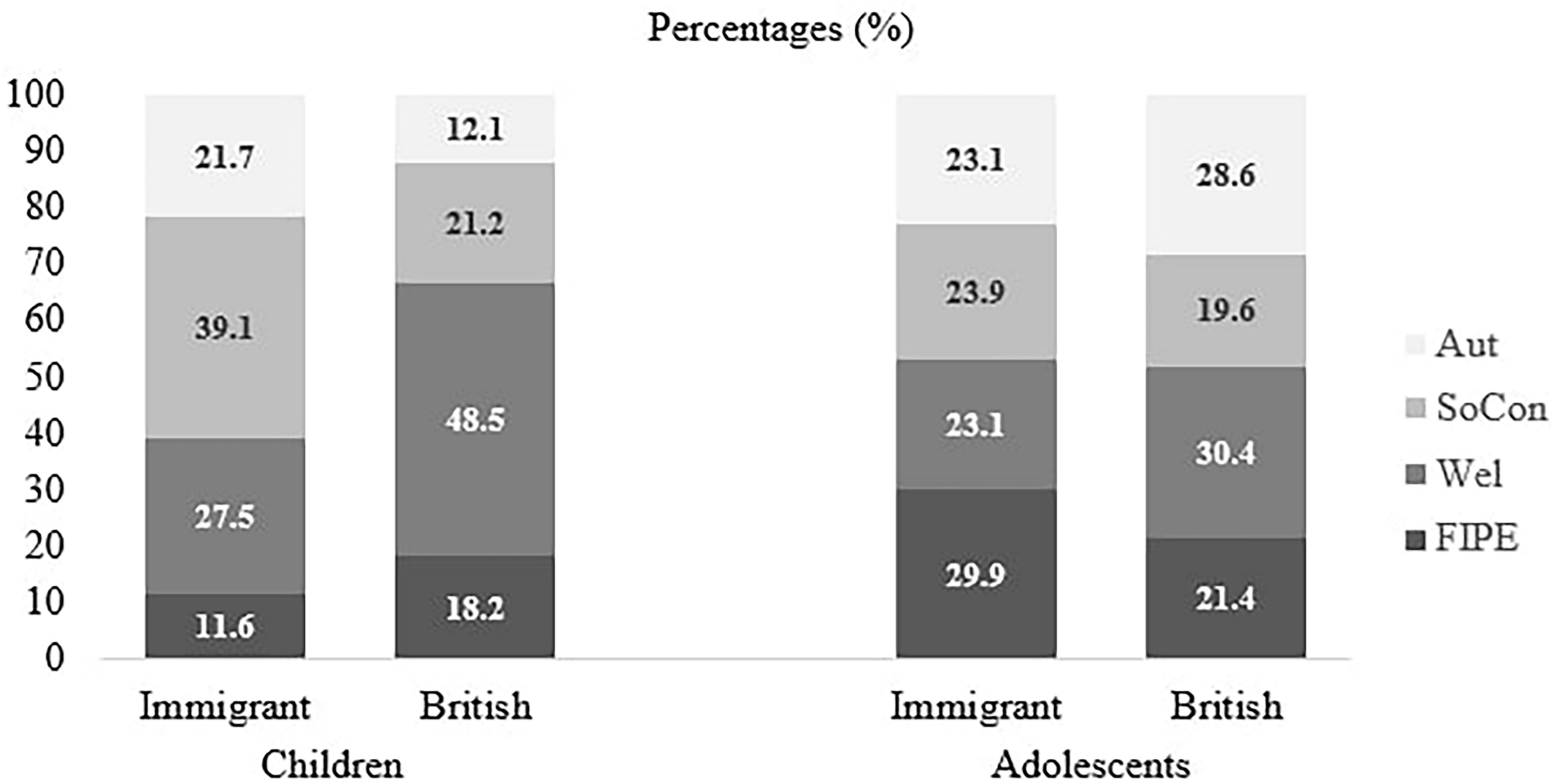
Percentages of participants reasoning of individual evaluation of challenger by exclusion condition and age group. FIPE, Fairness and Individual Rights and Prejudice and Equality; Wel, Welfare; SoCon, Social and Group Norms, Group Dynamics and Functions and Repercussions and Representation Management; and Aut, Autonomy. Numbers represent the percentages of participants within “Immigrant” and “British” conditions.

Lastly, “welfare” justifications were more likely to be used than “autonomy” justifications by children only in British exclusion condition, *p* = 0.044 (but not in immigrant exclusion condition, *p* = 0.839). For example, children who read about a British excluded peer provided justifications for their evaluations like “Because then he would not be lonely.” Contrary to children, adolescents’ justifications about “welfare” and “autonomy” did not differ in both conditions (see Figure 4 for the raw percentages for each category based on age and condition).

#### Perceived Group Evaluations of Challenger Reasoning

We conducted a multinomial logistic regression to examine participants’ reasoning about their evaluations of the group toward challenger across four categories: 1-Fairness, Rights, Prejudice, and Equality, 2-Welfare (of others), 3-Social and Group Norms, and 4-Group Dynamics/Functions Repercussions and Reputation Management. Exclusion condition (immigrant and British), age groups (children and adolescents), and group evaluation of challenger (categorical: okay versus not okay) were entered as factors. The model with all predictors was significant, *χ*^2^ (9, *N* = 234) = 21.65, Nagelkerke *R*^2^ = 0.10, *p* = 0.010. Both Pearson’s chi-square test [χ^2^ (12) = 3.61, *p* = 0.989] and Deviance chi-square [χ^2^ (12) = 4.26, *p* = 0.978] indicate good fit.

**Figure 5:**
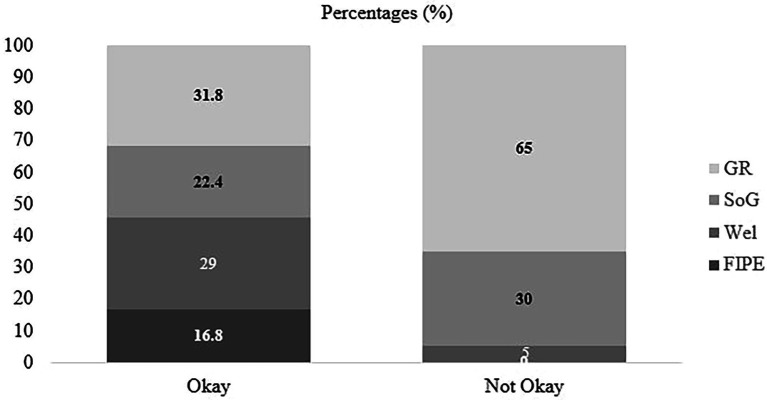
Percentages of participants reasoning of perceived group evaluation of challenger by their group evaluation judgments of challenger (categorized as okay versus not okay). FIPE, Fairness and Individual Rights and Prejudice and Equality; Wel, Welfare; SoG, Social and Group Norms; and GR, Group Dynamics and Functions and Repercussions and Representation Management. Numbers represent the percentages of participants within “okay” and “not okay” categories.

A significant main effect of perceived group evaluation was found, *χ*^2^ (3, *N* = 234) = 16.59, *p* < 0.001. More specifically, participants who reported that their group would evaluate challenger as not okay were more likely to attribute “social and group norms” (*p* < 0.001; e.g., “I think that because the group said that it was an British group and not any other countries; Alex betrayed us”) and “welfare” (e.g., “That they have been a bit nasty to both Deniz and Alex”; *p* < 0.001) than “fairness, rights and equality” compared to participants who reported that their group would evaluate challenger as okay (e.g., “It’s okay because Jamie deserves to be treated like everyone else, she is a normal human just like the rest of the group”). Further, participants who reported that their group would evaluate challenger as not okay were more likely to attribute “group dynamics/functions, repercussions and reputation management” (e.g., “Sam sounds as if she is a leader and the group may think it’s wrong to disagree with her; He might be thinking about Sam kicking him out of the group”) than “welfare” (*p* < 0.001) compared to participants who reported that their group would evaluate challenger as okay (e.g., “Because it is a nice thing to do and it might make Deniz very happy”; H5 was supported, see Figure 5 for the raw percentages).

## Discussion

The current study provided novel insights into how British children and adolescents evaluate ingroup challenger peers who object to social exclusion, especially when exclusion involves immigrant peers versus British peers as excluded peers. This study revealed that children and adolescents show different patterns in differentiating between their individual and group evaluations of an ingroup challenger across intergroup and intragroup contexts.

Children’s and adolescents’ ability to differentiate their own evaluation from the group’s perspective is one of the critical skills required to navigate complex intergroup situations (Mulvey et al., 2014). As we expected, our results showed that context (either intergroup or intragroup) shapes how children and adolescents differentiate between their individual and group evaluations. More specifically, as expected, when the newcomer peer was an immigrant both children and adolescents thought their peer group would evaluate the challenger significantly less positively than they would. This is in line with earlier studies that suggest that intergroup factors such as group membership (being ingroup or outgroup) are salient in school settings (Rutland and Killen, 2015; Brenick and Romano, 2016). Negative attitudes toward immigrants in the United Kingdom continue to rise in different social contexts including school settings, which can make immigration one of the salient intergroup contexts among children and adolescents (Blinder and Richards, 2020; Pavetich and Stathi, 2021). Thus, both children and adolescents might consider peer group norms more when determining their group’s evaluation of an ingroup challenger of intergroup exclusion involving an immigrant peer excluded peer.

In contrast, when the newcomer was a non-immigrant (i.e., British), only adolescents thought their peer group would evaluate the challenger significantly less positively than they would, and children did not differentiate between their individual and group evaluations. This is in line with previous studies documenting adolescents’ greater capacity to attend to both their group’s perspective and moral concerns compared to children (Mulvey et al., 2014). While adolescents’ reasoning frequencies were relatively similar across different justifications domains in both intragroup and intergroup social exclusion contexts, children’s reasoning justification was more unbalanced, especially in the intragroup context.

Regarding age-related patterns, our results showed that adolescents were more likely to evaluate the ingroup challenger positively in both their individual and group evaluations compared to children when the excluded peer was an immigrant. Adolescents might be more likely to think the underlying reason behind intergroup social exclusion is prejudice and discrimination since they are more aware of intergroup processes compared to children. In turn, compared to children, adolescents were more likely to be positive toward an ingroup challenger who stands up against racism and discriminatory tendencies and to think that their peers would be supportive of the ingroup challenger. This is also in line with some previous studies documenting increasing prosocial bystander responses with age (Mulvey et al., 2018; Yüksel et al., 2021) in an intergroup context. However, it should also be noted that there are studies suggesting reverse developmental age patterns in children’s and adolescents’ judgments and evaluations of social transgression. For example, Gönültaş and Mulvey (2021) showed that high school students were more likely to evaluate the bias-based bullying of immigrants as acceptable compared to middle school students. Although none of those studies examined age-related patterns in the context of bystander challenger evaluations, they still provide implications to show the complexity of developmental differences in children’s and adolescents’ judgments in an intergroup context. Our results also showed that when the excluded peer was British, adolescents’ individual evaluations were more positive than children’s, but children’s and adolescents’ group evaluations of the challenger did not significantly differ.

Our results also provide novel insights into participants’ reasoning behind their individual and group evaluations of ingroup challengers. As we expected, participants who evaluated the challenger as not okay were more likely to justify their evaluation using reasoning focused on social norms and group functioning than moral domains (e.g., fairness and welfare; e.g., “Because new students deserve the right to make friends; I think that because she might not have any friend and if we do not invite her then she is going to be really lonely”) compared to participants who evaluated the challenger as okay. Further, our results showed that children were more likely to refer to social and group norms than morality only when the excluded peer was an immigrant but not when the excluded peer was British. Similarly, children were more likely to use welfare justifications than autonomy only when the excluded peer was British but not when the excluded peer was an immigrant. This suggests that children’s reasoning justifications were more likely to differ based on the group membership of the excluded peers while adolescents were more likely to use similar justifications for their reasoning regardless of the group membership of excluded peers. In terms of reasoning judgments regarding the group evaluations, a similar pattern was observed based on participants’ evaluations. More specifically, participants who reported that their group would evaluate the challenger’s actions as not okay were more likely to reason using social conventional domain justifications than moral domain justifications compared to participants who reported that their group would evaluate the challenger’s actions as okay. Contrary to our predictions, group membership of the excluded peer and age did not relate to participants’ reasoning. It is possible that both children and adolescents are more likely to focus on group-related processes while providing justifications for their group perspective rather than focusing on group membership of excluded peers.

### Limitations and Future Directions

Despite novel insights, the current study has some limitations. First, as the study’s design was cross-sectional, it is difficult to infer the causality and to have a complete developmental picture. Thus, longitudinal studies would be helpful to explore further the mechanism behind age-related patterns. Further, we only examined the evaluation of challenger in middle childhood and adolescence. However, recent studies also investigated the infants’ evaluations and expectations about defensive and non-defensive puppets. For example, Geraci (2020) showed that 20-month-olds preferred the puppet that defended the victim puppet (“pushed by the aggressor puppet compared to the non-defensive puppet”). Further, Geraci and Surian (2021) examined 21-month-old infants’ expectations about punishing and rewarding a defensive puppet through the violation-of-expectation paradigm. They demonstrated that infants looked longer to the bystander puppet that punished the defensive puppet compared to the non-defensive puppet. They found reverse-looking patterns with the reward. These studies provide insights into early developmental patterns in evaluations of defenders in social contexts. Thus, future research could also examine evaluations of challengers in early childhood in the context of social exclusion.

Second, although we manipulated the group membership of the excluder across scenarios (Turkish, Australian, and British), we kept the group membership of challenger (British) and the excluder (British) as constant for the specific purpose of our study. However, future research should also examine how group membership of excluders and challengers might shape children’s and adolescents’ individual and group evaluations. Third, our findings are only limited to the immigration context in the United Kingdom. Although some previous studies provide similar evidence in some other contexts (e.g., gender-based), it is worth paying attention to the issue of contextual differences in different intergroup settings. Fourth, there is a possibility that participants could tend to align with others’ expectations or could have tried to answer in socially acceptable ways. However, we also wanted to acknowledge that this methodology has been used successfully in several studies to measure reasoning around intergroup biases in a manner that avoids social desirability (e.g., Mulvey and Killen, 2015; Rizzo et al., 2016). Often what is socially desirable is not crystal clear, since children and adolescents hear many comments from parents, teachers, and the media that are anti-immigrant and/or reflect ingroup preferences. Thus, it could be socially desirable to state that “our group is the most important” and show explicit biases against immigrants. In fact, in many of intergroup social exclusion studies, children and adolescents endorse ingroup biases and claims that other groups are “different” or not meritorious (e.g., Palmer et al., 2015; Mulvey et al., 2016, 2018; Gönültaş and Mulvey, 2021; Yüksel et al., 2021). Further, we also ensured our participants that any response could not linked back to the participant or schools and cannot be used to identify them individually within the data set. Lastly, although we involved open-ended “Why” questions to have an insight into the justifications of their judgments, we did not ask follow-up questions. Future research can examine participants’ reasoning about evaluating a challenger of intergroup social exclusion with a more comprehensive reasoning assessment approach, one that uses counterprobes and requests for evaluations of other hypothetical peers’ reasoning, which has been shown to be effective for providing multiple measures of reasoning responses (Rizzo et al., 2016).

### Conclusion

Addressing the factors that might encourage children and adolescents to challenge intergroup social exclusion, which can inform interventions are critical for a better future for youth and society. Our results show the importance of understanding how children and adolescents think and reason differently about bystander challengers in intergroup and intragroup exclusion contexts. In this study, adolescents, unlike children, readily expected that their group would evaluate the challenger more negatively than they would due to their advanced understanding of group dynamics. This understanding was only evident in children when the context made group identity and norms salient. Children’s reasoning behind their own evaluations of the challenger also differed from adolescents. Children, unlike adolescents, varied their reasoning more depending on the context, being more likely to reason about social processes than moral concerns only when the excluded peer was an immigrant. These findings suggest that children consider social and group norms when evaluating bystander challenging in “hot” or salient intergroup contexts, and interventions aimed at reducing exclusion of immigrants among children need to pay attention to the social and peer group norms that either support or challenge the exclusion of immigrants. Considering the current negative climate regarding immigrants in the United Kingdom and many other parts of the world, it is vital to develop strategies that focus on tackling the social exclusion of immigrant children and adolescents to promote inclusive school settings.

## Data Availability Statement

The original contributions presented in the study are included in the article/Supplementary Material, further inquiries can be directed to the corresponding author.

## Ethics Statement

The studies involving human participants were reviewed and approved by the University of Goldsmith. Written informed consent to participate in this study was provided by the participants’ legal guardian/next of kin.

## Author Contributions

SG made contributions to the data analysis, interpretation of data, and drafting of the manuscript. EK made contributions to the data analysis and interpretation of data and provided feedback to revise the manuscript critically for important intellectual content. AY made contributions to the acquisition of data and provided feedback to revise the manuscript critically for important intellectual content. SP made contributions to the design of the project and the acquisition of data and provided feedback to revise the manuscript critically for important intellectual content. LM and MK made contributions to design of the project and provided feedback for the manuscript. AR made contributions to the design of the project, the acquisition, analysis, and interpretation of data, and drafting of the manuscript, and revised the manuscript critically for important intellectual content. All authors contributed to the article and approved the submitted version.

## Funding

This research was funded by a grant from the Economic and Social Research Council (ES/R005540/1).

## Conflict of Interest

The authors declare that the research was conducted in the absence of any commercial or financial relationships that could be construed as a potential conflict of interest.

## Publisher’s Note

All claims expressed in this article are solely those of the authors and do not necessarily represent those of their affiliated organizations, or those of the publisher, the editors and the reviewers. Any product that may be evaluated in this article, or claim that may be made by its manufacturer, is not guaranteed or endorsed by the publisher.
