# Supplementary figures and images for: British Adolescents Are More Likely Than Children to Support Bystanders Who Challenge Exclusion of Immigrant Peers
Source: Front Psychol. 2022 Aug 8;13:837276. doi: 10.3389/fpsyg.2022.837276 (PMC9396375; doi:10.3389/fpsyg.2022.837276)

Supplementary Documents

Female group of friends


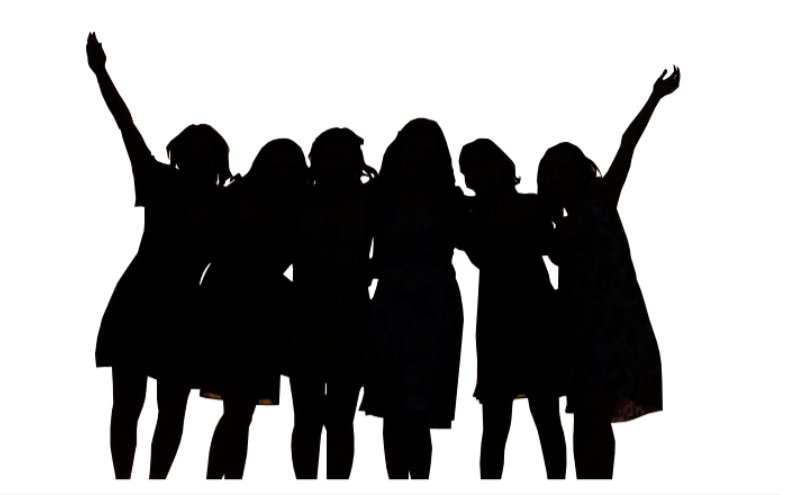


Male group of friends


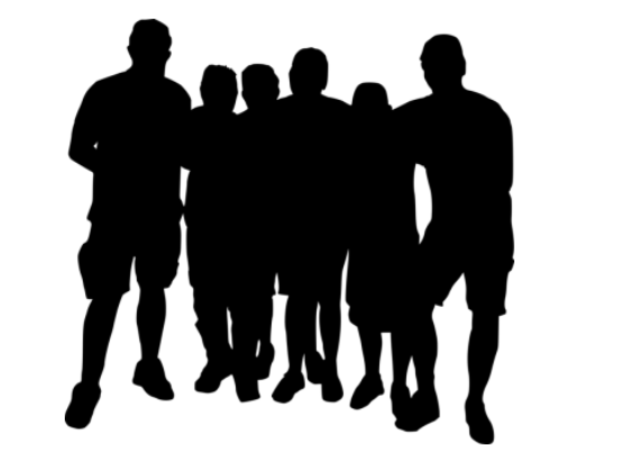

Supplement: Supplementary file 1 [file Data_Sheet_1.docx]
