# Supplementary material for: British Adolescents Are More Likely Than Children to Support Bystanders Who Challenge Exclusion of Immigrant Peers
Source: Front Psychol. 2022 Aug 8;13:837276. doi: 10.3389/fpsyg.2022.837276 (PMC9396375; doi:10.3389/fpsyg.2022.837276)
Supplement: Supplementary file 2 [file Data_Sheet_2.docx]

Supplementary Materials

(Female versions for all condition were presented).

1. **Turkish Condition**

This is a map of Britain. You live in England, which is part of Britain.

Are you British?

o Yes

o No

o I don't know

**-----------------------------------------------------------------------------------------**

We would like you to imagine that you are in the story and tell us what you think of what is happening. In the story, let's say that you are part of a group of friends who all live in England, which is in Britain. All your friends in this group were born here in Britain. Everyone in this group describes themselves as British

**-----------------------------------------------------------------------------------------**

Here is a map of the world. We have zoomed in to show you where British people are from.

We want to check you understand so far. Where were your friends in this story born?

o They were all born in Britain.

o They were all born in countries other than Britain.

o I don't know

Choose a name for your group of British friends:

________________________________________________________________

How much do you like being part of this group of British friends?

o No way

o Not really

o Not much

o Yes, a little

o Yes, a lot

o Yes, definitely

Your school has organised some after-school clubs.

Imagine that your group of friends has chosen to do an activity that involves cooking and baking food that is popular in Britain. Think of the types of food that you and your group might like to cook together and write some below:

______________________________________________________________

Imagine one week, there’s a new student who has come along to your group’s cooking club and wants to join in.

Deniz was born in Turkey. She recently moved from Turkey with her family to live in Britain.

We want to make sure you understand the story. Where was Deniz born?

o Britain

o Turkey

o Australia

Sam, who is in your group of friends, says to Deniz, "We don't want you to join our group because you are from somewhere else - you're different."

Alex is one of the friends in your British group. They disagree with Sam. Alex thinks that your group should invite Deniz to cook with them.

Imagine that Alex tells Sam that they think the group should invite Deniz to cook with them.

How OK or not OK was it for Alex to say that to Sam?

o Definitely not OK

o Not really OK

o Not much

o Yes, a little OK

o Yes, a lot

o Yes, definitely OK

Why do you think that?

________________________________________________________________

How OK or not OK does your group think Alex is, for telling Sam that Deniz should be invited to cook with the group?

o Definitely not OK

o Not really OK

o Not much

o Yes, a little OK

o Yes, a lot

o Yes, definitely OK

Why do you think that?

________________________________________________________________

1. **Australian Condition**

This is a map of Britain. You live in England, which is part of Britain.

Are you British?

o Yes

o No

o I don't know

**-----------------------------------------------------------------------------------------**

We would like you to imagine that you are in the story and tell us what you think of what is happening. In the story, let's say that you are part of a group of friends who all live in England, which is in Britain. All your friends in this group were born here in Britain. Everyone in this group describes themselves as British

**-----------------------------------------------------------------------------------------**

Here is a map of the world. We have zoomed in to show you where British people are from.

We want to check you understand so far. Where were your friends in this story born?

o They were all born in Britain.

o They were all born in countries other than Britain.

o I don't know

Choose a name for your group of British friends:

________________________________________________________________

Choose a symbol for your group of British friends.

How much do you like being part of this group of British friends?

o No way

o Not really

o Not much

o Yes, a little

o Yes, a lot

o Yes, definitely

Your school has organised some after-school clubs.

Imagine that your group of friends has chosen to do an activity that involves cooking and baking food that is popular in Britain. Think of the types of food that you and your group might like to cook together and write some below:

______________________________________________________________

Imagine one week, there’s a new student who has come along to your group’s cooking club and wants to join in.

Charlie was born in Australia. She recently moved from Australia with her family to live in Britain.

We want to make sure you understand the story. Where was Charlie born?

o Britain

o Turkey

o Australia

Sam, who is in your group of friends, says to Charlie, "We don't want you to join our group because you are from somewhere else - you're different."

Alex is one of the friends in your British group. They disagree with Sam. Alex thinks that your group should invite Charlie to cook with them.

Imagine that Alex tells Sam that they think the group should invite Charlie to cook with them.

How OK or not OK was it for Alex to say that to Sam?

o Definitely not OK

o Not really OK

o Not much

o Yes, a little OK

o Yes, a lot

o Yes, definitely OK

Why do you think that?

________________________________________________________________

How OK or not OK does your group think Alex is, for telling Sam that Charlie should be invited to cook with the group?

o Definitely not OK

o Not really OK

o Not much

o Yes, a little OK

o Yes, a lot

o Yes, definitely OK

Why do you think that?

________________________________________________________________

1. **British Condition**

This is a map of Britain. You live in England, which is part of Britain.

Are you British?

o Yes

o No

o I don't know

**-----------------------------------------------------------------------------------------**

We would like you to imagine that you are in the story and tell us what you think of what is happening. In the story, let's say that you are part of a group of friends who all live in England, which is in Britain. All your friends in this group were born here in Britain. Everyone in this group describes themselves as British

**-----------------------------------------------------------------------------------------**

Here is a map of the world. We have zoomed in to show you where British people are from.

We want to check you understand so far. Where were your friends in this story born?

o They were all born in Britain.

o They were all born in countries other than Britain.

o I don't know

Choose a name for your group of British friends:

________________________________________________________________

Choose a symbol for your group of British friends.

How much do you like being part of this group of British friends?

o No way

o Not really

o Not much

o Yes, a little

o Yes, a lot

o Yes, definitely

Your school has organised some after-school clubs.

Imagine that your group of friends has chosen to do an activity that involves cooking and baking food that is popular in Britain. Think of the types of food that you and your group might like to cook together and write some below:

______________________________________________________________

Imagine one week, there’s a new student who has come along to your group’s cooking club and wants to join in.

Jamie was born in Britain. She recently moved here with her family from somewhere else in Britain..

We want to make sure you understand the story. Where was Jamie born?

o Britain

o Turkey

o Australia

Sam, who is in your group of friends, says to Jamie, "We don't want you to join our group because you are from somewhere else - you're different."

Alex is one of the friends in your British group. They disagree with Sam. Alex thinks that your group should invite Charlie to cook with them.

Imagine that Alex tells Sam that they think the group should invite Charlie to cook with them.

How OK or not OK was it for Alex to say that to Sam?

o Definitely not OK

o Not really OK

o Not much

o Yes, a little OK

o Yes, a lot

o Yes, definitely OK

Why do you think that?

________________________________________________________________

How OK or not OK does your group think Alex is, for telling Sam that Charlie should be invited to cook with the group?

o Definitely not OK

o Not really OK

o Not much

o Yes, a little OK

o Yes, a lot

o Yes, definitely OK

Why do you think that?

________________________________________________________________
